# A System of Practical Medicine, Comprised in a Series of Original Dissertations

**Published:** 1840-12-05

**Authors:** 


### MEDICAL EXAMINER.

DEVOTED TO MEDICINE, SURGERY, AND THE COLLATERAL SCIENCES.

No. 49.] PHILADELPHIA, SATURDAY, DECEMBER 5, 1840. [Vol. III.

### BIBLIOGRAPHICAL NOTICE.

*A System of Practical Medicine, comprised in a series of Original Dissertations.* Arranged and edited by Alexander Tweedie, M. D., F.R.S., Fellow of the Royal College of Physicians, Physician to the London Fever Hospital, and to the Foundling Hospital, etc. Vol. I. Philadelphia: Lea & Blanchard. 1840.

The "System of Practical Medicine" is the first series of a work entitled the "Library of Medicine," now publishing in England, under the direction of Dr. Tweedie. The design of the "Library" is to supply the medical literature of Great Britain with "a comprehensive system of medicine, embodying a condensed, yet ample view, of the present state of the science." In our own country, the want of such a work, at least on the practice of medicine, has been for some time much felt, for, however valuable some of our elementary treatises on the subject may be, they are far from affording that complete yet condensed information which it is so desirable to meet with in consulting a work of the kind. The first series of the work before us, or that comprising the system of Practical Medicine, has been completed in England in five volumes. Two of these volumes have been republished in this country, and a third is in the press. The first volume, to which our present notice will be confined, treats of fevers, and diseases of the skin. The work opens with a pathological introduction, from the pen of Dr. Symonds, occupying sixty pages. In this short space the author has given us a most masterly summary of the prominent facts and principles of general pathology. He has presented to us a brief, yet comprehensive sketch of the characters of congestion, inflammation, the different morbid secretions, heterologous formations, &c. calculated to afford the reader definite conceptions of the nature of these processes, as far as facts enable us to understand them. Such an account of the elementary forms of morbid action is particularly important to the student during the earlier part of his studies, and the want of it is often very embarrassing to him.

The subject of inflammation is separately and much more fully treated of in the following dissertation, and that it is ably and impartially handled, is sufficiently guaranteed by the fact that the dissertation in question is from the pen of Dr. Alison.

We are next presented with an account of the "general doctrines of fever," and of "continued fever," for which we are indebted to Dr. Christison. This gentleman is an advocate of the primary or essential nature of fever. With his general train of reasoning in support of this opinion, we perfectly coincide. Whilst he admits on the one hand that the essentialists have neglected too much the local affections which occur in fever, he thinks, on the other, that the non-essentialists have exaggerated their importance. However much the essentialists may have neglected these local affections, this neglect is no necessary consequence of the doctrine of the primary nature of fever. Its enlightened advocates should be as anxious as any others, in the treatment of a case, to diminish or dissipate local determinations, and never, for a moment, to lose sight of the local lesions which modern investigations have discovered. On the other hand, the advocates of the doctrine that fever is secondary and dependant upon inflammation, are necessarily led to measure the value of depletion and other remedies, by the influence they are calculated to exert upon the local affection, and consequently to exaggerate the importance of some, and depreciate that of other therapeutic agents. The difference in the effects of remedies, in fevers and inflammation, is, in our opinion, one of the strongest proofs of the essentially different nature of these two classes of diseases, and might with advantage have been added by Dr. C. in his summary of arguments. Continued fever he describes under the three forms of synocha, synochus, and typhus. Dothenenteritis, or the typhoid fever of Louis, he considers as a secondary affection, occasionally occurring in the course of true typhus, though at the same time he admits that it may have an independent existence. By thus confounding together two distinct diseases, viz., typhus and typhoid fever, and describing both under the general head of continued fever, Dr. C. has necessarily been led into considerable confusion, both as regards facts and reasonings, since what is perfectly true of one disease may not be so of the other. With this exception it strikes us that the dissertation in question is exceedingly well drawn up, and well worthy of the high reputation of its author. The American student should recollect, however, that it is mainly applicable to true typhus, and not to typhoid, or the common continued fever of this country.

Plague, intermittent, remittent, and yellow fevers have been assigned to Dr. Shapter, infantile remittent to Dr. Locock, and hectic fever to Dr. Christison. The dissertation of Dr. Locock on infantile gastric remittent fever is particularly worthy of attention. The account of small pox is drawn up by Dr. Gregory; that of measles, as also of scarlet fever, by Dr. G. Burrows. Next to these comes the history of puerperal fevers, in which the pen of Dr. Locock again comes into play. This gentleman has, independent of private practice, had vast opportunities of becoming practically acquainted with the subject, having been for nearly eighteen years attached to a very large lying-in hospital. He thinks that the fever of puerperal women is not always the same, and that it is not invariably characterised by any uniform local lesion. He hence objects to the terms peritonitis, &c. and prefers that of puerperal fevers, which compromises no opinion as regards their nature.

The concluding chapter on diseases of the skin, and occupying nearly a hundred pages, is written by Dr. Schedel, of Paris, a name well known to those who are at all conversant with this branch of medical science.

Our limits of course will not allow of our attempting an analysis of the statements and opinions of the different writers in the volume before us. The several dissertations of which it is composed are eminently practical in their tendency, embodying in the main a full and impartial account of what is known in reference to the various subjects treated of, unobscured by the mass of absurd speculations which so often disfigure medical productions. It is a work which should be in the hands of every student and young practitioner, for nowhere else, we think, will he find, in the same space, the same amount of useful information in reference to the history and treatment of disease. The American edition is exceedingly well got up, on good paper, and with a larger type than that employed in the English edition.

